# Evidence of excretion of Schmallenberg virus in bull semen

**DOI:** 10.1186/1297-9716-45-37

**Published:** 2014-04-04

**Authors:** Claire Ponsart, Nathalie Pozzi, Emmanuel Bréard, Virginie Catinot, Guillaume Viard, Corinne Sailleau, Cyril Viarouge, Julie Gouzil, Martin Beer, Stéphan Zientara, Damien Vitour

**Affiliations:** 1 LNCR - Laboratoire National de Contrôle des Reproducteurs, 13 rue Jouet, 94704 Maisons-Alfort, France; 2 UMR1161 Virologie ANSES-INRA-ENVA, 23 avenue du Général de Gaulle, 94704 Maisons-Alfort, France; 3 Institute of Diagnostic Virology, Suedufer 10, 17493 Greifswald-Insel Riems, Germany

## Abstract

Schmallenberg virus (SBV) is a novel orthobunyavirus, discovered in Germany in late 2011. It mainly infects cattle, sheep and goats and could lead to congenital infection, causing abortion and fetal abnormalities. SBV is transmitted by biting midges from the *Culicoides* genus and there is no evidence that natural infection occurs directly between ruminants. Here, we could detect SBV RNA in infected bull semen using qRT-PCR (three bulls out of seven tested positive; 29 positive semen batches out of 136). We also found that highly positive semen batches from SBV infected bulls can provoke an acute infection in IFNAR^-/-^ mice, suggesting the potential presence of infectious virus in the semen of SBV infected bulls.

## Introduction, methods and results

In November 2011, a new virus was identified in Germany in a pool of blood samples from clinically affected dairy cows using a metagenomic approach [1]. This new virus called the Schmallenberg virus (SBV) belongs to the family *Bunyaviridae*, genus *Orthobunyavirus*, presenting sequence similarities with the Simbu serogroup viruses [2]. So far, SBV RNA has been detected in cattle, sheep and goats, whereas antibodies have also been detected in bison, roe deer, red deer, moufflon and alpacas (OIE Technical Facsheet, 2012). Cattle with acute infection may present mild symptoms such as a drop in milk yield, fever, and diarrhea, but clinical signs are not systematically observed. The viraemic stage is very short (1 to 6 days; [1]). The virus seems to be at least transmitted by midges belonging to the *Culicoides* genus [3,4]. As other viruses of the Simbu serogroup, typical malformations designated as “arthrogryposis hydranencephaly syndrome” (AHS) have been reported. Fetuses present torticollis and severe arthrogryposes (i.e. ankylosis and tendon shortening) combined with hydranencephaly and hydrocephalus. The central nervous system may show extreme deformations with porencephaly or hydranencephaly [5]. Altogether, the clinical picture is very similar to that of infections with Akabane virus [6,7]. The timing of infection of the dam during pregnancy for developmental defects of the fetus to occur seems critical and could be analogous to the Akabane virus. This period is about 4–8 weeks of pregnancy in sheep, and about 8–14 weeks in cattle [6,8]. Akabane virus could not be detected in semen collected from viraemic bulls following an experimental infection [9]. Intra-uterine inoculation of Akabane virus in cattle at the time of artificial insemination did not result in clinical disease but most animals developed viraemia. Virus could not be recovered from nasal or vaginal swabs but was isolated from a number of tissues, including the reproductive tract (ovaries, uterine) and associated lymph nodes from cows slaughtered up to day 7 after intrauterine inoculation [8]. All pregnant cows at term delivered healthy calves [8]. Gard et al. [10] used semen from bulls naturally infected with Simbu serogroup viruses to inoculate sheep. Although four animals seroconverted, the possibility that these animals were infected naturally by vectors could not be excluded.

Data regarding the role of semen on the transmission of SBV and other Simbu serogroup viruses are limited. Recently, the Friedrich-Loeffler-Institut (FLI) analyzed the semen of 94 bulls with a known SBV-antibody status for the presence of SBV genome, with 26 positive semen batches from 11 bulls and subsequent SBV RNA C_t_-values ranging from 26 to 37. According to the authors, the findings could indicate that SBV behaves differently from Akabane virus in relation to semen contamination [11,12].

To ensure safe trade of cattle germplasm, semen must be collected and processed at approved and supervised semen collection centres, obtained from animals whose health status ensures there is no risk of spread of any animal disease through artificial insemination. The objectives of the study presented here were to detect the potential excretion of SBV in semen of naturally seroconverting bulls and to establish the duration and infectivity of SBV in relation to serological status.

Seven bulls, 1 to 5 years of age, with no detectable SBV neutralizing antibodies prior to the beginning of the study were selected with the following criteria: SBV seroconversion observed between September 2011 and December 2012, due to a naturally occurring infection during this period, and the most complete semen production batches including at least 14 ejaculates, collected from 4 weeks before to 4 weeks after the first positive SBV ELISA result (Table 1).

**Table 1:** Characteristics of SBV seroconverting bulls

**Bull (N#)**	**Year of birth**	**Breed**	**N# of tested semen batches**	**Date of first ejaculate**	**Date of last ejaculate**	**SBV antibody levels**	
						**Day-0**	**Day-28**
1	2006	Ho	37	13/10/2011	26/03/2012	22/11/11 (201%)	10%
2	2010	Ho	18	18/06/2012	19/09/2012	21/08/12 (165%)	1%
3	2010	No	15	18/06/2012	29/08/2012	21/08/12 (91%)	2%
4	2010	Ho	18	18/06/2012	19/09/2012	21/08/12 (142%)	1.6%
5	2010	Ho	18	20/06/2012	17/09/2012	21/08/12 (67%)	2.6%
6	2010	No	14	18/06/2012	27/08/2012	21/08/12 (124%)	0.6%
7	2011	No	16	03/08/2012	03/12/2012	14/08/12 (52%)	1.4%

Day-0: day of first positive SBV ELISA result. Day-28: S/P values 4 weeks before the first positive ELISA result. Ho = Holstein; No = Normand.

SBV ELISA results are expressed as S/P values and are given in percentages.

For each bull, the presence of SBV specific antibodies was tested monthly in serum using an indirect ELISA kit (ID Screen® Schmallenberg virus Indirect ELISA kit, IDvet, Montpellier, France) according to the manufacturer’s instructions [13]. The results were expressed as S/P values (S/P = OD sample/OD positive control)*100). Samples were considered as negative when S/P < 50%, positive when S/P > 60% and doubtful when S/P was between 50 and 60%. Date of seroconversion was defined as the date giving the first positive ELISA result (Table 1).

Semen from SBV ELISA positive bulls was collected under field conditions, from two different locations with a semen collection agreement, once or twice a week, diluted using a commercial extender containing egg yolk (bulls 2 to 7) or a home-made extender without egg yolk (bull 1), conditioned in 0.25 mL straws after 4 h of equilibration and frozen using a glycerol-based classical procedure [14]. When an SBV positive ELISA bull was identified, a retrospective study was conducted on semen batches collected before and after SBV seroconversion to search for the presence of SBV RNA by one-step real time RT-PCR (qRT-PCR). A validation study was previously performed in our laboratory, in order to quantify the detection limit of this method from fresh sperm and diluted straws (not shown). The qRT-PCR used was previously developed and validated by the FLI [1]. RNA extraction was automated using King Fisher equipment (Thermo Fisher).

Then, the presence of infectious SBV in semen was appreciated by inoculating IFNAR^-/-^ mice with SBV RNA positive semen samples. Semen replicates (each 100 μL) from ejaculates were injected subcutaneously into the neck scruff of three or four adult IFNAR^-/-^ mice. After 4 days, EDTA blood samples were collected and tested by SBV qRT-PCR. The presence of SBV-specific antibodies was detected by ELISA (IDvet) 2 to 3 weeks post-inoculation (pi). Mice were observed daily for clinical signs and weights were assessed from day 3 until day 7 pi. All experiments were performed under the guidelines of the European Community (86/609) and were approved by the common ethical review committee from ANSES-INRA-ENVA, Maisons-Alfort, France (reference number: 13-021).

**Figure 1:**
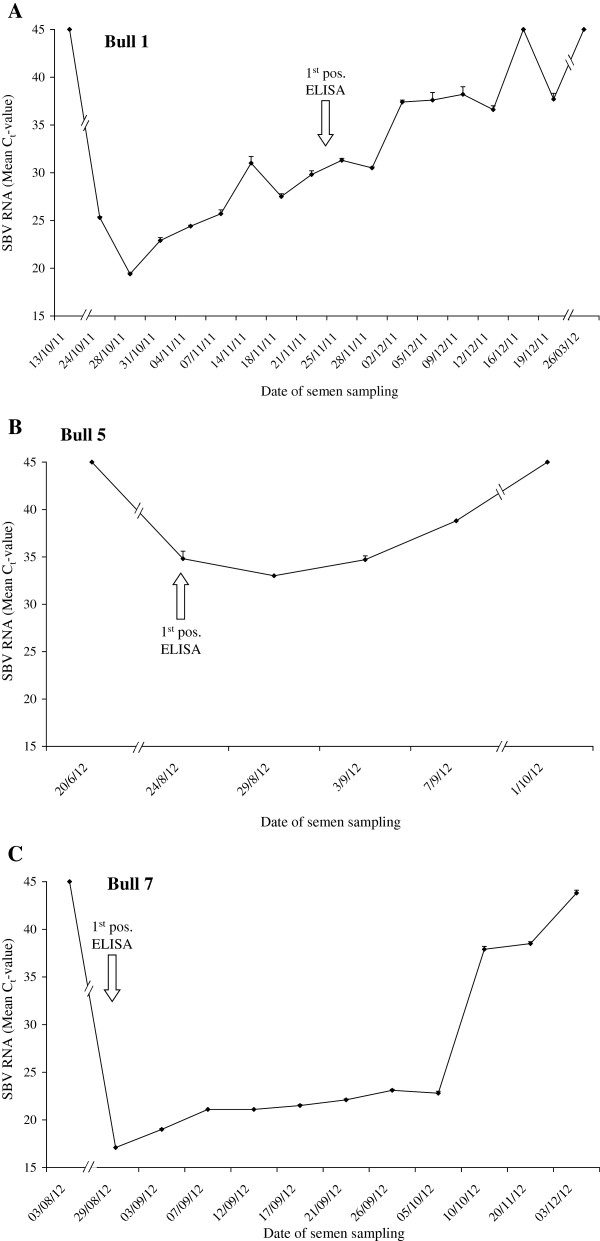
**Evolution of SBV RNA levels in semen straws of bulls 1 (A), 5 (B) and 7 (C).** Kinetic follow-up of SBV RNA qRT-PCR was performed on samples of 10 μL of three straws (one single ejaculate). The results are expressed as mean C_t_ values ± standard errors of triplicates. The first positive SBV ELISA test is depicted on the graph.

All seven bulls presented a SBV seroconversion by ELISA (S/P values > 60%) as the first positive result (Table 1), but one bull was considered as doubtful (S/P = 52%) and seroconversion was confirmed one week later (bull 7, confirmed on 21/08/2012, with S/P value = 78%). No SBV RNA was detected in four (bulls 2, 3, 4, 6) out of the seven seroconverted bulls before and following seroconversion. However, three bulls from different breeds (Holstein or Normand) were found SBV RNA positive on more occasions using qRT-PCR (Figure 1).

The time period with detectable viral RNA in sperm ranged from 2 weeks (bull 5) to almost 3 months (bull 7) following the first positive serological sample with highly variable C_t_-values (17.1 to 38.5), indicating that the virus can be detected up to 3 months upon infection. Interestingly, bulls 1 and 7 displayed a continual increase of SBV RNA C_t_ values throughout the follow-up until viral RNA became undetectable. In bull 1, one negative batch (16/12/2011) was followed by a positive test (19/12/2011), which could correspond to intermittent excretion as previously reported but also to the presence of a very low amount of SBV RNA molecules in the semen closed to the detection limit of the qRT-PCR method [11]. Bull 5 presented a shorter and lower excretion pattern, starting 4 days after the first positive ELISA test and lasting 14 days only, with high C_t_-values (C_t_ > 30).

The presence of infectious SBV in semen was then appreciated by inoculating IFNAR^-/-^ mice which are susceptible to the infection [15]. In all in vivo experiments depicted below, inoculated mice showed no clinical signs and their weights were unchanged during the follow-up. First, 100 μL of semen replicates from one SBV RNA positive ejaculate (bull 7 sampled on 29/08/2012; C_t_ = 17.1) were injected subcutaneously to three adult mice. After 4 days, EDTA blood samples were collected and were found SBV RNA positive (C_t_-values ranged between 23 and 28.9) (Table 2). They all seroconverted within 3 weeks following infection. In a second in vivo assay, four groups of four IFNAR^-/-^ mice were inoculated with the same semen batch (collected the 29/08/2012) as well as semen samples collected before or after this date (Table 2). Again, all four mice inoculated with the semen batch from the 29/08/2012 displayed high SBV RNA levels in blood 4 days pi (C_t_-values ranged between 20 to 26) and were SBV ELISA positive 3 weeks pi. Inoculation of the semen from 07/09/2012 induced low SBV RNA and antibody levels in two out of four mice, while SBV RNA and antibodies remained undetectable in all mice upon inoculation with the semen from 17/09/2012. Interestingly, the semen collected the 24/08/2012 induced positive SBV C_t_ value in only two mice but without detectable SBV antibody by ELISA. It is noteworthy that all mice with no detectable SBV RNA 4 days pi also had no detectable SBV antibody in serum, suggesting that a viraemia seems to be required to induce a humoral response upon inoculation with naturally SBV RNA positive semen. This was in favor of the presence of a replicative virus in the inoculum. Finally, positive SBV blood samples collected from mice at day 4 pi with semen from 29/08/2012 during experiment 2 were pooled (Inoculum A) and subsequently diluted 1 to 10 or 1 to 100 in MEM medium. Two groups of four mice were inoculated with 100 μL of these two blood preparations (experiment 3). Among all inoculated animals, one mouse was found SBV qRT-PCR and ELISA positive when inoculated with the IFNAR blood diluted 1/10 (Table 2). This indicates that the SBV RT-PCR positive IFNAR^-/-^ blood collected at 4 days pi with the semen from 29/08/2012 (experiment 2) and inoculated to new IFNAR^-/-^ mice (experiment 3), contained infectious material at probably low levels but sufficient to induce SBV viraemia and seroconversion in at least one mouse.

**Table 2:** **Detection of SBV RNA in IFNAR**^**-/- **^**mice inoculated with 100 μL of semen from bull 7 (Experiments 1 and 2) or with SBV RNA positive blood from IFNAR**^**-/- **^**mice (Experiment 3)**

**Inoculum**	**Exp. N°**	**Mice N°**	**C**_**t **_**inoculum**	**C**_**t **_**blood (4 days pi)**	**ELISA results**
Bull 7 (24/08/2012)	2	8587	**33.6**	**36.7**	Neg
		8585		Neg	Neg
		8589		**24.0**	Neg
		8581		Neg	Neg
Bull 7 (29/08/2012)	1	7728	**17.1**	**28.9**	**Pos**
		8216		**23.5**	**Pos**
		8410		**23.0**	**Pos**
	2	8583	**19.7**	**21.7**	**Pos**
		8849		**20.0**	**Pos**
		8582		**25.6**	**Pos**
		8588		**26.0**	**Pos**
Bull 7 (07/09/2012)	2	8827	**22.3**	**31.5**	**Pos**
		8571		Neg	Neg
		8847		Neg	Neg
		8856		**37.0**	**Pos**
Bull 7 (17/09/2012)	2	8621	**23.1**	Neg	Neg
		8709		Neg	Neg
		8853		Neg	Neg
		8630		Neg	Neg
Inoculum A (1/10)	3	8631	**22.0**	**22.1**	**Pos**
		8634		Neg	Neg
		8858		Neg	Neg
		8629		Neg	Neg
Inoculum A (1/100)	3	8688	**25.0**	Neg	Neg
		8682		Neg	Neg
		8848		Neg	Neg
		8625		Neg	Neg

qRT-PCR results from 10 μL of straws or blood sampled from IFNAR^-/-^ mice, 4 days pi.

Bold data correspond to samples with a confirmed detection of SBV RNA (Ct inoculum and blood) or positive ELISA results.

All together, these results strongly suggest that semen batches collected the 29/08/2012 and the 07/09/2012 contained infectious SBV.

## Discussion

The present findings confirm that bull semen can contain infectious SBV and that semen keep infectivity even after bull seroconversion, as evidenced by in vivo experimental infection using the IFNAR^-/-^ mice model. Different excretion patterns were observed in three out of seven seroconverted bulls. Two animals (bulls 1 and 7) produced high levels of SBV RNA in their semen (C_t_ < 20) whereas low SBV RNA amounts were detected in bull 5. In the four other SBV ELISA positive bulls, no SBV RNA was detected. These results suggest a large variability in the excretion of SBV in semen of naturally infected bulls. Particular patterns in semen viral RNA were characterized in two different breeds, with a sustained and prolonged SBV genome in semen batches, as recently reported [11,12]. However, bulls 1 and 7 presented lower SBV RNA C_t_-values (C_t_ < 20) compared to bulls followed by the FLI, presenting SBV RNA C_t_-values ranging from 26 to 37 [11]. No clear intermittent excretion was observed in this study. The variability of the presence of SBV RNA in semen over time seemed to indicate that the virus genome may disappear progressively in some cases (Bulls 1 and 5), whereas it persisted in a more stable and intensive pattern in bull 7. Although SBV RNA C_t_-values remained low (C_t_ < 25) for a long time following seroconversion in bull 7, the presence of infectious virus seemed to be limited, with only two batches (collected the 29/08/2012 and 07/09/2012) able to induce viraemia and seroconversion in IFNAR^-/-^ mice. It is interesting to note that this variability was observed despite the low number of animals studied. It may be hypothesized that SBV RNA variable profiles might be affected by “dose-effect” factors, or by different susceptibilities to SBV infection.

These results also show that SBV seroconversion observed in IFNAR^-/-^ mice after inoculation of SBV qRT-PCR positive semen batches correlates with a detectable viraemia 4 days pi. In this study, no clinical sign was observed and weight was unchanged in inoculated mice. This was reminiscent of previous findings obtained with infectious bovine blood inoculated to IFNAR^-/-^ mice [16]. However, these results are distinct to previous observations published by Wernike et al. who found that IFNAR^-/-^ mice inoculated with SBV lost weight and became sick [15]. This difference may be attributed to poorly infectious virus or virus present in low amounts that is not sufficient to induce clinical signs. This might also rely on distinct genetic background or different mice ages, both resulting in different SBV susceptibilities. In addition, a titer dependence cannot be excluded.

It is actually difficult to compare behavior between SBV and other worldwide orthobunyaviruses, as Akabane, Aino or Cache Valley viruses in semen, considering the facts that i) limited scientific data are available regarding semen shedding, ii) a quite low proportion of SBV-seropositive bulls present positive RT-PCR results, iii) the virus detection methods developed for semen need to be highly sensitive to detect the RNA genome (specific extraction protocols have been developed recently for SBV) iv) culture or isolation may not be sensitive enough for this group of RNA viruses, which could explain previously reported Akabane negative culture results [9]. Akabane virus was isolated from a number of tissues, including the reproductive tract (ovaries, uterine) and associated lymph nodes from cows slaughtered up to day 7 after intrauterine inoculation [17]. The risk for any orthobunyavirus to be transmitted through semen should be reconsidered and assessed using sensitive diagnostic tools as qRT-PCR before delivering any scientific recommendation regarding the absence of transmission via insemination.

In conclusion, this study reports the presence of SBV RNA in bull semen that can be detected for several months after natural infection. It also suggests for one particular highly positive SBV RNA semen batch the putative presence of infectious virus that, however, could not be isolated. Indeed, the risk of virus transmission from SBV-positive semen needs to be further assessed including in vivo infection approaches with intra-uterine inoculation of SBV-positive semen.

## Abbreviations

qRT-PCR: (quantitative) Real time reverse transcription-polymerase chain reaction; SBV: Schmallenberg virus.

## Competing interests

The authors declare that they have no competing interests.

## Authors’ contributions

CP structured the article and prepared, along with NP, MB, EB and DV, the draft of the manuscript. CV, DV, JG, CS and EB performed in vivo experiments. EB, CV, VC, GV did the qRT-PCR and ELISA assays. MB and SZ reviewed the paper before submission. All authors read and approved the final manuscript.
